# The Evolutionary Loss of Paternal Care Is Associated With Shifts in Female Life‐History Traits

**DOI:** 10.1002/ece3.70497

**Published:** 2025-04-23

**Authors:** Colby Behrens, Sarah Young, Eric Arredondo, Anne C. Dalziel, Laura K. Weir, Alison M. Bell

**Affiliations:** ^1^ Department of Evolution, Ecology and Behavior School of Integrative Biology, University of Illinois at Urbana‐Champaign Urbana Illinois USA; ^2^ Department of Biology St. Mary's University Halifax Nova Scotia Canada; ^3^ Program in Ecology, Evolution and Conservation University of Illinois Urbana‐Champaign Urbana Illinois USA; ^4^ Carl R. Woese Institute for Genomic Biology University of Illinois Urbana‐Champaign Urbana Illinois USA; ^5^ Program in Neuroscience University of Illinois Urbana‐Champaign Urbana Illinois USA

**Keywords:** clutch size, life history, paternal care, reproductive effort

## Abstract

Parental care can increase the fitness of parents through increased offspring survival but can also reduce reproductive output by limiting time and energy allocated to additional mating opportunities. The evolutionary origin of parental care is often associated with shifts in life‐history traits (e.g., high investment in few, large offspring, slow offspring growth), but little is known about whether the evolutionary *loss* of care is associated with reciprocal shifts in the same life‐history traits. Here, we capitalize on the divergence of parental care between ecotypes of three‐spined stickleback (*Gasterosteus aculeatus*) to test for associations between parental care and life‐history traits. While males from most stickleback populations provide care, an unusual “white” ecotype has recently lost paternal care. We found support for the hypothesis that the evolutionary loss of paternal care is associated with shifts in female life‐history traits; relative to females of the ecotype with paternal care, females of the white ecotype that lack paternal care produced clutches with a similar overall mass and a greater number of smaller eggs, despite their smaller body size, suggesting lower per‐offspring investment. We did not detect an ecotypic difference in embryonic development rate, metabolic rate, or offspring age at hatching, contrary to the ‘safe harbor hypothesis’. These results support the theory that behavioral traits such as parental care co‐evolve with other life‐history traits and highlight opportunities for future study of the underlying causal mechanisms.

## Graphical abstract

Parental care has evolved independently multiple times and is often associated with shifts in additional life‐history traits, such as increased egg size, but what happens when care is lost? Here, we show that an unusual population of sticklebacks that evolved a loss of typical paternal care also exhibit changes in maternal life‐history traits, including decreased egg size. These results highlight that male and female life‐history traits evolve in a coordinated way during both evolutionary gains and losses of parental care in one sex.

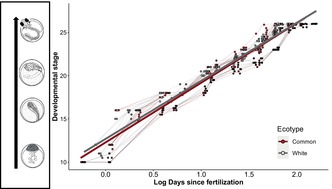

## 1 Introduction

Parental care, while relatively rare, is phylogenetically widespread and integral to reproductive success in many species of animals (Royle, Smiseth, and Kölliker 2012). Care provided during early development can improve overall offspring quality and survival and has important implications for the fitness of both the parents and offspring (Royle, Smiseth, and Kölliker 2012; Clutton‐Brock 1991). Parental care is also energetically costly and can leave parents vulnerable to threats such as predation (Clutton‐Brock 1991; Klug, Alonzo, and Bonsall 2012), potentially decreasing survival and future reproductive success.

Consequently, trade‐offs involved with care can lead to variation in parental care both within and among taxa. Some variation in care can be attributed to factors such as parental body condition, resource availability, and parental age (Carlisle 1982; Gross and Sargent 1985; Saino et al. 2000). Likewise, parental investment is often carefully balanced between current offspring and future mating opportunities (Trivers 1974; Clutton‐Brock 1991). Differences in care can also evolve due to sexual selection and conflict. Individuals may alter their investment in care after assessing prospective mates for quality, condition, and potential parental investment (Trivers 1972; Westneat and Sargent 1996; Sheldon 2000; Chapman et al. 2003; Alonzo 2012), and these alterations can occur both before and after mating (Kraak and Van Den Berghe 1992). These complex relationships can cause parental care to co‐evolve with a variety of traits related to courtship, mating, and offspring investment (Behrens et al. 2024). Additionally, considering the multitude of conflicts and shared fitness effects among members of a biological family, it is not surprising that maternal, paternal, and offspring traits often co‐evolve to maximize fitness based upon traits of other family members (Parker, Royle, and Hartley 2002). For example, experimental evolution studies with burying beetles have shown that offspring traits (e.g., body size, density, and morphology) evolve rapidly in response to a reduced parenting regimen (Schrader et al. 2017; Jarrett Benjamin, Evans et al. 2018) and that offspring can rapidly adapt to the loss of care through other means such as sibling cooperation (Rebar et al. 2020).

Theory and data suggest that the evolution of parental care is associated with a shift in a suite of life‐history traits, including relatively larger and fewer offspring, greater gametic investment, prolonged offspring dependence, and slower growth (reviewed in Chapter 2 Royle, Smiseth, and Kölliker 2012; Clutton‐Brock 1991; Gross and Sargent 1985; Sargent, Taylor, and Gross 1987; Kolm and Ahnesjö 2005; Smith and Fretwell 1974). Indeed, the evolution of parental care is associated with larger egg size in fishes and this effect is exacerbated in species with paternal care (Benun Sutton and Wilson 2019). A proximate explanation for this observation is that oxygen limitations in aquatic systems make larger eggs with a lower surface to volume ratio more likely to experience hypoxia than smaller eggs if mass‐specific metabolic rates are similar (Van Den Berghe and Cross 1989; Pettersen et al. 2018, but see Einum, Hendry, and Fleming 2002). The ubiquity of oxygen‐provisioning and embryo fanning behaviors in aquatic environments offers indirect support for this hypothesis (Jones and Reynolds 1999). Also, interspecific comparisons find that larger eggs have a longer developmental period (Pauly and Pullin 1988; Gillooly et al. 2002), though this is not always found at the intraspecific level (Einum, Hendry, and Fleming 2002).

From an ultimate perspective, parental care may be associated with larger eggs and prolonged offspring development to increase survival in a more dangerous juvenile stage and provide a ‘safe harbor’ (Shine 1989). Observations in a variety of taxa support this idea; for example, experimental removal of fathers resulted in earlier hatching in glass frogs (Delia, Ramírez‐Bautista, and Summers 2014). Similarly, burying beetle larvae that did not receive care evolved to hatch earlier (and more synchronously; Jarrett Benjamin, Rebar et al. 2018). According to the ‘safe harbor hypothesis’ (Shine 1989), a change in either egg size, embryo development time, or level of parental care would favor the co‐evolution of complementary changes in the other traits (Shine 1978, 1989; Nussbaum 1987; Kolm and Ahnesjö 2005).

Therefore, theory and data strongly support the hypothesis that the evolution of parental care is associated with a shift in life‐history characteristics (i.e., fewer, larger eggs and longer development time; Shine 1978; Kolm and Ahnesjö 2005). However, little is known about whether the evolutionary *loss* of care is associated with a shift in life‐history traits in the opposite direction (Udu, Bonsall, and Klug 2022), which could provide further support for theory linking the evolution of parental care and life‐history strategies (Shine 1978; Kolm and Ahnesjö 2005). Here, we capitalize on a recent evolutionary loss of paternal care in three‐spined sticklebacks (*Gasterosteus aculeatus*) to explore how these traits co‐evolve when parental care is lost.

Male three‐spined sticklebacks typically provide extensive parental care that is necessary for offspring development and survival (e.g., fungus removal, protection from predators, Wootton 1976). Females release eggs into a male‐constructed nest, then males fertilize the eggs and aerate the embryos through fanning for approximately 5–6 days (Wootton 1976). Males continue to guard the offspring for about one week after hatching (Wootton 1976). However, there is wide variation in the type and quality of parental care among individuals and populations of sticklebacks (Borg 1985; Stein and Bell 2015).

In Nova Scotia, Canada, there is an ecotype of three‐spined stickleback that provides an extremely reduced form of care, named the “white” stickleback because of the male's bright dorsal coloration during the breeding season (hereafter designated with “NC” for non‐caring; Blouw and Hagen 1990; Blouw 1996). The white stickleback ecotype often lives in sympatry with the common stickleback (hereafter designated with “C” for caring), which provides parental care and displays typical blue/green/brown nuptial body coloration (Blouw and Hagen 1990; Blouw 1996; Behrens et al. 2024). Immediately after fertilization, white (NC) males remove the eggs from their nest and disperse them to surrounding algae and substrate, after which they resume nesting and begin a new mating cycle, providing more time for additional matings (Blouw 1996; Jamieson, Blouw, and Colgan 1992). This divergence in parental care between these populations has a heritable basis (Blouw 1996), and population genomic analyses suggest the white (NC) population recently diverged (< 12,000 years) from the sympatric common (C) population of sticklebacks and that there is ongoing gene flow between ecotypes (Samuk 2016).

There is abundant evidence that female three‐spined stickleback life‐history traits such as egg size and number can diverge rapidly, e.g., between benthic‐limnetic populations (Baker et al. 2005), anadromous‐freshwater populations (Baker, Heins, and Baum 2019; Kurz et al. 2016; Karve, Baker, and Von Hippel 2013; Oravec and Reimchen 2013), among freshwater populations (Baker, Foster, Heins, Bell, and King 1998; Baker, Heins, King, and Foster 2011; Baker, Rasanen, Moore, and Hendry 2013) and between anadromous populations (Kume 2011). Previous studies on the common‐white ecotypes have suggested that the white (NC) ecotype sexually matures at a smaller size and remains smaller than the common form throughout its lifetime (Blouw 1996). Preliminary evidence also suggests that the ecotypes may differ in a number of life‐history traits, such as development rate and clutch size (Grant 1993). Therefore, their recent divergence and the extreme differences in reproductive strategies between these two ecotypes make them a compelling system to test theory about how maternal, paternal, and offspring life‐history traits co‐evolve with the evolutionary loss of parental care. We hypothesized that the loss of paternal care is associated with reduced per‐offspring investment by females (smaller eggs and larger clutches) and faster embryonic development, supported by a higher mass‐specific embryonic metabolic rate.

## 2 Materials and Methods

### 2.1 Animal Collection and Maintenance

In June 2018 and 2022, adult common (C) and white (NC) stickleback fish were collected from two primarily allopatric sites in eastern Canada using minnow traps and dip nets to measure female life‐history traits and embryonic development. Common (C) fish were collected from Cherry Burton Road, New Brunswick, CA (N 46° 01.516′ W 64° 06.150′), while white (NC) fish were collected from Canal Lake, Nova Scotia, CA (44°29′54.0"N 63°54′09.1"W) under the Department of Fisheries and Oceans Maritime (#343930) and Gulf (SG‐RHQ 18–022 and 19–008) Region Scientific Collection permits to ACD. Through June and July, 2023, stickleback were collected from two additional Nova Scotia sites to measure embryo metabolic rates. Adult commons (C) were collected from Antigonish landing (45°38′03.3"N 61°57′01.8"W) and Lawrencetown Beach (44°38′44.0"N 63°20′28.8"W) and white (NC) fish were collected from Lawrencetown Beach. We note that it is difficult to unequivocally label sites as “allopatric” or “sympatric” because the presence of white and common ecotypes varies temporally and spatially across a site.

Fish (F0 generation) were transported to Saint Mary's University in Halifax, Nova Scotia, where they were kept in holding tanks with a salinity of 10 ppt, a temperature of 18°C–21°C, and a 16:8 (L:D) photoperiod. In 2018 and 2022, fish were then flown to Champaign, Illinois where they were housed at the University of Illinois in Urbana‐Champaign. Fish were separated by population and kept in large aquaria with a water salinity of 10 ppt and a 16:8 (L:D) photoperiod at 20° C. All fish were fed a daily *ad libitum* diet of blood worms, *Mysis* and brine shrimp, and Cyclop‐eez. Wild‐caught fish had reached sexual maturity but were of unknown age. All fish were measured for standard length after arriving in the laboratory. The F0 fish were then bred and the F1 offspring were reared under similar conditions in the laboratory until maturity, at which point they were measured for standard length and F2 experimental embryos were generated as described below. All collections, housing conditions, and experiments were approved by the Saint Mary's University Animal Care Committee and University of Illinois Institutional Animal Care and use Committee.

### 2.2 Female Life‐History traits

To quantify egg size, clutch size, and reproductive effort (clutch mass), 14 clutches were generated through natural fertilizations (*n* = 7 common and *n* = 7 white) between lab‐reared F1 stickleback in 2019. A male stickleback was paired with a unique gravid female from the same ecotype and allowed to court and mate. The female was measured for standard length (head to caudal peduncle) and weight before being added to the male's tank. If a mating occurred, the female was weighed again and marked through dorsal or pectoral spine‐clipping, while the male was removed and sacrificed for additional experiments. Clutch mass (reproductive effort) was estimated as the difference in female weight immediately before and after spawning (Bell, Trapp, and Keagy 2018). Eggs were removed from the tank, the total number of eggs was counted, and five eggs from each clutch were physically measured to the nearest hundredth of a millimeter using digital calipers to estimate average egg size within a clutch.

### 2.3 Embryonic Development Rate

To measure embryonic development rate, 34 clutches of embryos (*n* = 14 common and *n* = 20 white) were generated through artificial fertilizations between lab‐reared stickleback in 2023. Gravid females were gently squeezed to release their eggs into a petri dish. Testes, dissected from sacrificed males, were macerated to release sperm into a Ginzburg Ringer's solution, which was then pipetted onto the eggs to fertilize them. Afterwards, embryos were incubated in mesh‐lined cups over a bubbling airstone following standard protocols (Day, Pritchard, and Schluter 1994) and were measured for embryonic development rate.

Each day after fertilization, the artificially fertilized embryos were removed from their incubators in the morning and evening (6–9 h gap), submerged in shallow water, and observed under a dissecting microscope. Dead embryos and fungus were removed to prevent clutch failures. Environmental variables, particularly temperature, can have a large effect on development rate (Pauly and Pullin 1988), so temperature, salinity, and dissolved oxygen were measured daily using a ProSolo YSI probe. These measures were consistent (20°C, 10 ppt salinity, and 8 mg/L oxygen saturation) over the course of the experiment.

Embryonic development rate in fishes is often estimated as days to hatch. However, this is a coarse‐grained metric that does not capture potential variation in the timing of important developmental transitions. Therefore, we measured embryonic development from fertilization to hatching by observing embryos under a dissecting microscope twice a day and tracked stickleback‐specific morphological landmarks in embryonic development (Swarup 1958). Briefly, the developmental process begins at stage 1 when fertilization occurs. As the heart, eyes, and other major organs develop the embryos progress to stage 26 when larvae hatch. Because embryos do not develop uniformly within a single clutch, the stage of each embryo in a clutch was identified and percentages at each stage were recorded to track diversity within a clutch. An average score for each clutch for each observation period was calculated, as developmental progression among eggs often varied by 1–3 stages. We recorded the day the clutch hatched (stage 26) and fish were removed from the incubators.

### 2.4 Embryo Metabolic Rate

In 2023, wild‐collected F0 fish were held at Saint Mary's University and fed a daily *ad libitum* diet of blood worms and Mysis shrimp. Fish were on a 16:8 (L:D) photoperiod and temperature was maintained at 18°C–21°C. The F0 generation was used for *in vitro* crosses and the resulting 15 clutches (*n* = 8 common and *n* = 7 white) were reared in holding tanks with a salinity of 10 ppt and methylene blue added to the water and kept in the same L:D conditions as F0 fish. Within the holding tanks, individual clutches were kept together in small plastic‐sided containers with mesh bottoms. Embryos were not fed for the duration of experiments as they still had their yolk‐sac.

Embryonic oxygen consumption rate was measured as a proxy for whole organism metabolic rate. Oxygen consumption rates of common (C) and white (NC) sticklebacks were taken when the embryos were three days post fertilization using a Microplate Respirometry System (Loligo Systems, Viborg, Denmark). Oxygen sensors were calibrated prior to each trial according to manufacturer specifications. Embryos were individually placed into 18 of the 24 80 μL wells in the glass microplate, leaving the remaining 6 wells empty to record background oxygen consumption concurrently. The wells were sealed to be air‐tight using adhesive PCR plate seals (BioRad, Mississauga, ON, Canada) and a silicon sheet (Loligo Systems, Viborg, Denmark). The plate was enclosed under opaque casing to prevent unnecessary external disturbance. Sealed and encased microplates were placed within a water bath of constantly flushing 18°C, 10 ppt water to maintain constant temperature within the wells. The water bath setup was placed on a shaker (Thermo Scientific, Compact Digital Mini Rotator, Waltham, MA, USA) which gently mixed water within the microplate to disperse oxygen evenly throughout each well and ensure accurate oxygen readings during the two hour trial. Following each metabolic rate trial, embryo diameter was measured (i.e., diameter of the chorion) to standardize metabolic rates by size (μm). Oxygen consumption rate was calculated as the slope of the oxygen decline divided by the individual embryo's chorion diameter (μmol O_2_ μm^−1^). Individual oxygen consumption rates were averaged across individuals within a clutch.

### 2.5 Statistical Analysis

We analyzed the standard length of 119 wild‐caught and 49 lab‐raised individuals via a linear model that included ecotype, rearing environment (wild‐caught or lab‐reared), sex, and an ecotype by rearing environment interaction as fixed effects. The function ‘emmeans’ was used to perform pairwise contrasts between ecotypes and p‐values were adjusted for multiple testing via the Bonferroni method. We analyzed egg size, clutch size (number of eggs), and clutch mass of *n* = 14 lab‐reared females (7 common (C), 7 white (NC)), the development rate of *n* = 34 clutches (from *n* = 14 common (C) females and *n* = 20 white (NC) females) and the metabolic rate of *n* = 15 clutches (from *n* = 8 common (C) and *n* = 7 white (NC) females). Days to hatch and female standard length were normally distributed and analyzed using linear models with the ‘lm’ function to test for associations with other traits (e.g., egg size, clutch size), and un‐paired t‐tests were used to test for differences between the ecotypes. Prior to analysis, clutch size and clutch mass were log‐transformed. To test for an effect of ecotype on egg size, we applied a linear mixed effect model with clutch as a random effect using the ‘lmer’ function in the package ‘lme4’ (Bates et al. 2007). The effects of female body size on life‐history traits (egg size, clutch size) were tested with linear regression, and size‐corrected residuals were analyzed. To test for an effect of ecotype on embryonic metabolic rate, we applied a linear mixed effect model. We fit metabolic rate as a response variable, ecotype as a fixed effect, and family as a random effect. Development rate was non‐linear, necessitating a log transformation of embryo age. To test for an effect of ecotype and age on embryo development, we applied a linear mixed model. We fit developmental stage as a response variable, post fertilization age (log‐transformed), ecotype, and their interaction as fixed effects, and clutch identity as a random effect. We also fit a simpler model that used overall development rate (stage/age) as a response variable, ecotype as a fixed effect, and clutch as a random variable, which yielded similar results. Data were analyzed using R version 4.0.3 (R Development Core Team 2011). All graphs were generated using the package ‘ggplot2’ (Wickham 2011).

## 3 Results

### 3.1 Female Life‐History Traits

**FIGURE 1:**
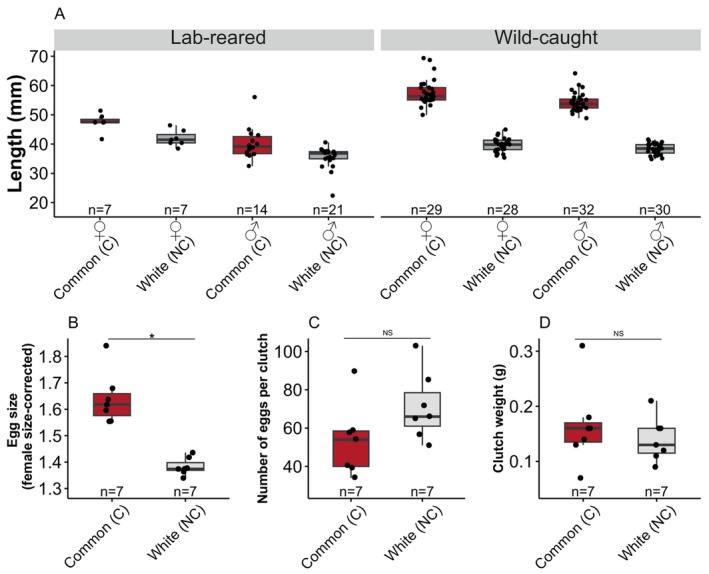
Female white (NC) three‐spined stickleback are smaller and produce additional, smaller eggs than female common (C) stickleback. Box plots (interquartile range with median) are shown in all figures with each data point representing a different individual. A. Adult commons (C) were consistently larger than adult whites (NC) when reared in the lab or collected from the wild. B. Females of the common (C) ecotype produced larger eggs than females of the white (NC) ecotype. As egg size was significantly correlated with female size (Figure S1 B), residuals of egg size versus female body length for an individual egg are presented. C. Females of the white (NC) ecotype produced marginally larger clutches (number of eggs) compared to females of the common (C) ecotype. D. Clutch mass does not differ between ecotypes.

Adult commons (C) were consistently larger than adult whites (NC), females were larger than males, and wild‐caught individuals (age unknown) were larger than one year old lab‐reared individuals (Figure 1A, Table 1). Females from the white (NC) ecotype produced eggs that were 18% smaller than common (C) eggs (Figure S1 A; *t* = 6.534, df = 12, *p* < 0.001). Egg size was positively associated with female standard length (Figure S1 B; *β* = 0.03, *R* ^2^ = 0.6248, *N* = 14, *p* < 0.001) and body mass (*β* = 0.40, *R* ^2^ = 0.6925, *N* = 14, *p* < 0.001), and egg‐bearing females of the common (C) ecotype were longer (Figure 1A; *t* = 5.189, *p* = < 0.001) and heavier (*t* = 6.49, df = 12, *p* < 0.001) than egg‐bearing females of the white (NC) ecotype. To compare maternal investment across ecotypes, we corrected for differences in egg size attributable to maternal size by regressing egg size on female standard length and analyzing the residuals. We chose to regress on female standard length, not mass, because length is less susceptible to short‐term changes due to feeding and length and mass were highly correlated (*β* = 11.72, *R* ^2^ = 0.8787, *N* = 14, *p* < 0.001). A linear mixed effect model, using ecotype as a fixed effect and clutch as a random effect, showed that common (C) eggs were still larger than white eggs even after correcting for the larger female body lengths of common (C) mothers (Figure 1B; *F*_1,14_ = 4.89, *p* = 0.044).

**TABLE 1:** Linear model results testing for the effect of sex, rearing environment, ecotype, and the rearing by ecotype interaction on adult body size.

	df	MS	*F*	*P*
Sex	1	922.9	72.76	**< 0.001**
Rearing	1	1886.0	148.70	**< 0.001**
Ecotype	1	7713.2	608.15	**< 0.001**
Rearing:Ecotype	1	1145.2	90.30	**< 0.001**
Error	163	12.7		

Females from the white (NC) ecotype produced marginally larger clutches (more eggs) before controlling for female body size (Figure 1C; *t* = −2.016, df = 12, *p* = 0.067), and significantly larger clutches when female body size was taken into account (Figure S2 A; *t* = −3.920, df = 12, *p* = 0.002), though clutch size (egg number) was not significantly correlated with female body size (Figure S2 B; *β* = −0.029, *R* ^2^ = 0.133, *N* = 14, *p* = 0.199). Clutch mass (reproductive effort) was not correlated with female body size (Figure S3; *β* = −0.007, *R* ^2^ = 0.005, *N* = 14, *p* = 0.805) and did not differ between the ecotypes (Figure 1D; *t* = 0.566, df = 12, *p* = 0.582). Clutch size and egg size were also negatively associated (Figure S4; *β* = −0.287, *R* ^2^ = 0.368, *N* = 14, *p* = 0.021), suggesting that larger clutches comprise smaller eggs.

### 3.2 Embryonic Development and Metabolic Rates

**FIGURE 2:**
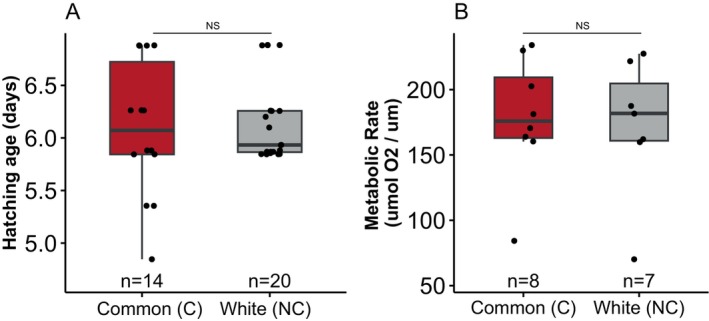
Ecotypes do not differ in time to hatch or mass‐specific metabolic rates. Box plots (interquartile range with median) are shown in all figures. A. Ecotypes did not differ in age of hatching. B. Common (C) and white (NC) embryos do not differ in metabolic rate.

**FIGURE 3:**
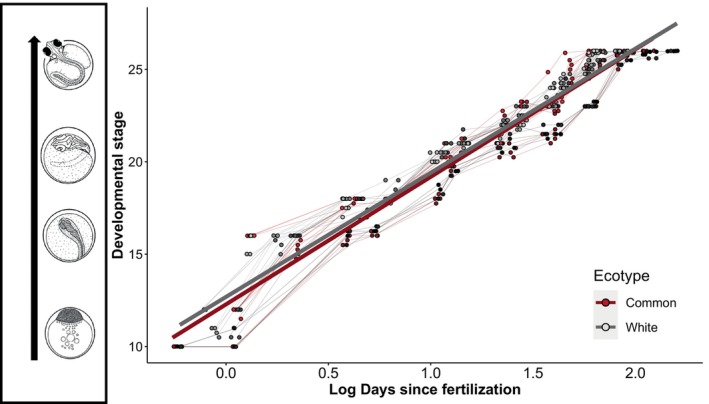
Common (C) and White (NC) embryos do not differ in development rate. Developmental stage of embryos of the Common (C) (red, *n* = 14 clutches) and White (NC) (gray, *n* = 20 clutches) ecotype as a function of age (days since fertilization). The corresponding drawings next to the Y axis are from Swarup (1958). Each data point represents the average developmental stage of a clutch and lines track their development over time. Data points and lines are colored separately for each clutch. Major trendlines represent the development rate of each ecotype. Days since fertilization was log‐transformed for statistical analysis. Hatching occurs at developmental stage 26 (day 6–7).

Latency to hatch of either the first (*t*‐test: common μ = 6.09, SD = 0.65, *n* = 14; white μ = 6.16, SD = 0.40, *n* = 20; *t*_32_ = −0.370, *p* value = 0.714; Figure 2A) or the last (*t*‐test: common μ = 6.72, SD = 0.77, *n* = 14; white μ = 7.13, SD = 0.89, *n* = 20; *t*_32_ = −1.412, *p* value = 0.168) embryo in a clutch did not differ between the ecotypes. The two ecotypes also showed similar mass‐specific metabolic rates at three days post‐fertilization (LMM *F*_1,9.6_, < 0.01, *p* = 0.98; Figure 2B). We detected a significant effect of age on embryonic development but found no evidence for an effect of ecotype (Figure 3, Table 2).

**TABLE 2:** Linear mixed effect model results testing for the effect of age, ecotype, and their interaction on embryonic developmental stage.

	df	MS	*F*	*p*
Log(age)	1, 413.94	7266.0	14326.09	**< 0.001**
Ecotype	1, 58.79	0.6	1.25	0.267
Log(age):Ecotype	1, 413.94	0.7	1.47	0.226

## 4 Discussion

Parental care has evolved independently in multiple lineages of animals, and there is tremendous diversity in the specific behavioral manifestations of care. Despite this diversity, the evolution of parental care is typically associated with similar life‐history patterns (e.g., few, large offspring; Klug, Alonzo, and Bonsall 2012). Theory and phylogenetically informed comparisons among species offer some insights into the ultimate mechanisms underlying these patterns, e.g. via parental investment theory (Klug, Alonzo, and Bonsall 2012). Empirical studies of the relationship between care and life history have focused on the evolutionary *origins* of care; far less attention has been given to its loss, in part because losses are less common in nature (Udu, Bonsall, and Klug 2022). In this study, we provide strong support for theory linking the evolution of parental care and life‐history strategies by showing that the relationship between care and life history works both ways, and across the two sexes; reduced parental investment by fathers, i.e., the loss of paternal care, was associated with a concomitant reduction in parental investment by mothers in individual offspring.

Egg size is often used as a proxy for female investment (Shine 1978; Levitan 1996; Kume 2011; Summers, McKeon, and Heying 2006) and relative to the common (C) ecotype, females of the white (NC) ecotype produce smaller eggs, suggesting that they invest less per individual egg. This finding matches theoretical expectations (Shine 1989; Kolm and Ahnesjö 2005) and patterns observed in other systems (Bagarinao and Chua 1986; Clutton‐Brock 1991). However, we note that the observed difference in egg size was relatively small, and geometric egg size is not always a reliable estimate of egg energy content (McEdward and Morgan 2001). Future studies should investigate differences in female investment in more detail by quantifying yolk size, carotenoids, lipid content, and/or protein content (McEdward and Morgan 2001). In addition to producing smaller eggs, there was suggestive evidence that female whites produce larger clutches, consistent with the familiar tradeoff between egg size and clutch size (Roff 1993), and with the hypothesis that changes in paternal care are associated with shifts in the partitioning of female reproductive investment. Moreover, both wild‐caught and lab‐reared whites were smaller than commons, indicating that the adult size difference is not solely due to differences in environment or age of wild populations, and consistent with previous studies (Blouw and Hagen 1990; Samuk 2016; Haley, Dalziel, and Weir 2019). More generally, the difference in adult body size suggests that divergence in parental care may be associated with a suite of additional life‐history traits including growth and age and size at maturity.

The Safe Harbor Hypothesis (Shine 1989) predicts that an increase in parental care is associated with a slower offspring development rate (Klug, Alonzo, and Bonsall 2012). In contrast to this hypothesis, we found that white (NC) embryos do not develop or hatch more quickly than their common (C) counterparts when reared in the same environment, contrary to previous findings in this system (Grant 1993). We also did not detect a difference in hatching rate, matching intraspecific studies in other species (Einum, Hendry, and Fleming 2002), or mass‐specific metabolic rate, supporting the finding that embryonic development rates are similar. Together, these data suggest that the white (NC) ecotype produces smaller, but metabolically and developmentally similar embryos. It is possible that under more natural conditions differences in development rate among ecotypes might occur. In particular, the larger common (C) embryos are predicted to be more susceptible to hypoxia due to their overall higher metabolic rates and oxygen diffusion limitations due to a lower surface area to volume ratio compared to the smaller white (NC) embryos. In this situation, a reduction in development rate and oxygen consumption might result if parental fanning (Van Iersel 1953; Head, Fox, and Barber 2017; Fox, Head, and Barber 2018) cannot maintain sufficient oxygenation of the embryos.

Altogether, these results support the hypothesis that the evolutionary loss of care is associated with an overall change in female and offspring life history. An outstanding question is whether the shift in female life‐history strategy preceded the loss of care or vice versa. Comparative work on the origin of care in amphibians suggests that larger eggs evolved before parental care (Summers, McKeon, and Heying 2006). If large egg size preceded the evolution of care in sticklebacks, then we might expect eggs to remain large when care is lost, which is not what was observed in this study. This result could be interpreted as support for the idea that behavioral traits (parental care) drive the evolution of other traits (Benowitz et al. 2018), but other selective forces may have acted to limit egg size (e.g., oxygen limitation when paternal fanning does not occur; Van Den Berghe and Cross 1989; Einum, Hendry, and Fleming 2002). Conversely, it is also possible that reduced female per‐offspring investment or that reduced offspring dependence caused males to provide less care (Hale and Travis 2012). Disentangling the causal factors and the co‐evolutionary interactions in the stickleback system is difficult as the causes of divergence between the caring and non‐caring ecotypes are likely multifaceted and reflect numerous selective factors. The ecotypes likely differ in life‐history strategies, sexual selection, sex ratios, coloration, and ecological drivers (e.g., nesting preferences and predation rates; Blouw 1996; Behrens et al. 2024) and have partially nonoverlapping breeding seasons, all of which could contribute to divergence, potentially in a mutually reinforcing manner (Kokko and Jennions 2008). Moreover, the evolutionary origin and loss of parental care is predicted to be rife with conflict and coadaptation among family members (Parker, Royle, and Hartley 2002), which makes it difficult to tease apart causal factors.

The pair of common (caring) and white (non‐caring) stickleback ecotypes offer several advantages for understanding the evolution of changes in parental care, namely natural variation between two recently diverged forms of the same species. The recent (< 12,000 years ago) divergence and potentially ongoing gene flow among ecotypes offer an opportunity to study the early stages of adaptation, and their natural variation provides numerous opportunities to understand the ecological drivers and context of parental care divergence. Further, the ecotypes can be crossed to generate fully fertile F1 hybrid offspring (Behrens et al. 2024), and the availability of genomic resources (Jones et al. 2012; Glazer et al. 2015) opens up the possibility of identifying the genetic, neural, and molecular mechanisms which contribute to divergence in reproductive strategies. Additional priorities for future work include determining if females of the white (NC) ecotype have a shorter inter‐spawning interval relative to females of the common (C) ecotype, collecting additional life‐history data (e.g., sex‐ and age‐specific death rates, lifespan and growth rate), and quantifying differences in ovarian fluid, which holds the clutch together but might be less “sticky” in whites (Grant 1993). Finally, it will be fascinating to know if the loss of care consistently evolves in parallel with life‐history traits across multiple, independent evolutionary losses of care in this species (Borg 1985; Samuk 2016).

## Author Contributions

**Colby Behrens:** conceptualization (equal), data curation (equal), formal analysis (equal), visualization (equal), writing – original draft (equal). **Sarah Young:** data curation (equal), writing – review and editing (equal). **Eric Arredondo:** data curation (equal), writing – review and editing (equal). **Anne C. Dalziel:** conceptualization (equal), writing – review and editing (equal). **Laura K. Weir:** conceptualization (equal), writing – review and editing (equal). **Alison M. Bell:** conceptualization (equal), funding acquisition (equal), writing – original draft (equal).

## Conflicts of Interest

The authors declare no conflicts of interest.

## Supporting information


Figure S1.
Figure S2.
Figure S3.
Figure S4.

## Data Availability

Data have been archived in the Dryad Digital Repository at the following link: https://doi.org/10.5061/dryad.zs7h44jhq.
