# Supplementary material for: The Evolutionary Loss of Paternal Care Is Associated With Shifts in Female Life‐History Traits
Source: Ecol Evol. 2025 Apr 23;15(4):e70497. doi: 10.1002/ece3.70497 (PMC12015637; doi:10.1002/ece3.70497)

**SUPPLEMENTAL MATERIAL**

**Figure S1.** A) Common (C) females produce larger eggs than white (NC) females. B) Egg size is significantly associated with female body length.


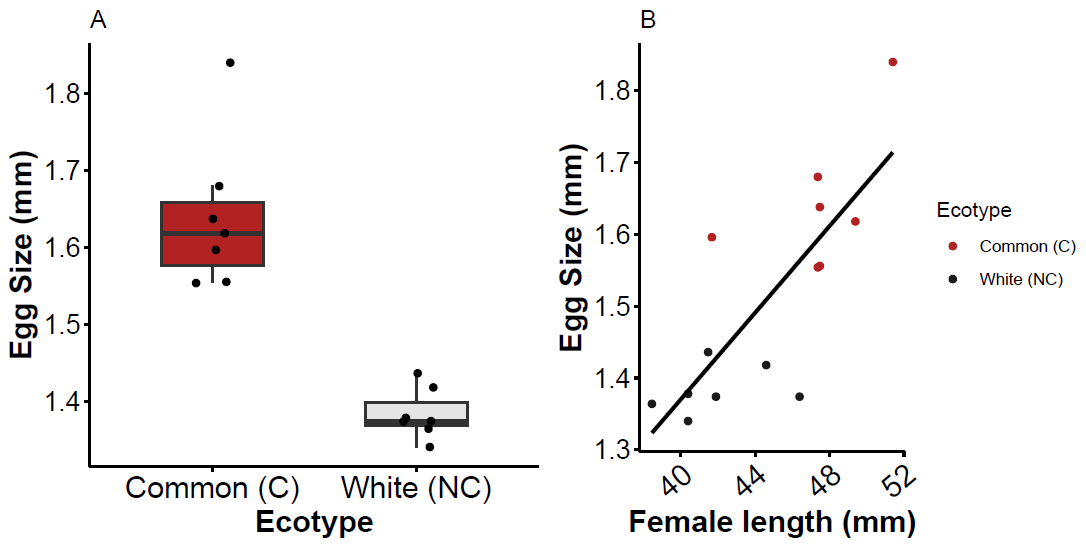


**Figure S2.** A) When corrected for female body size, common (C) females produce significantly fewer eggs within a clutch than white (NC) females. B) The number of eggs in a clutch is not associated with female body length.


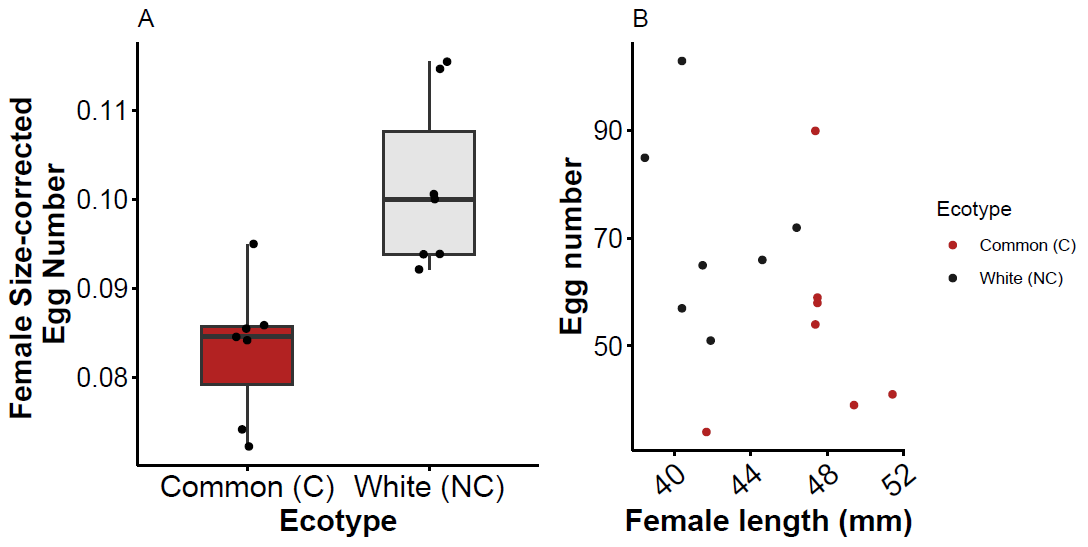


**Figure S3.** Clutch mass is not associated with female length.


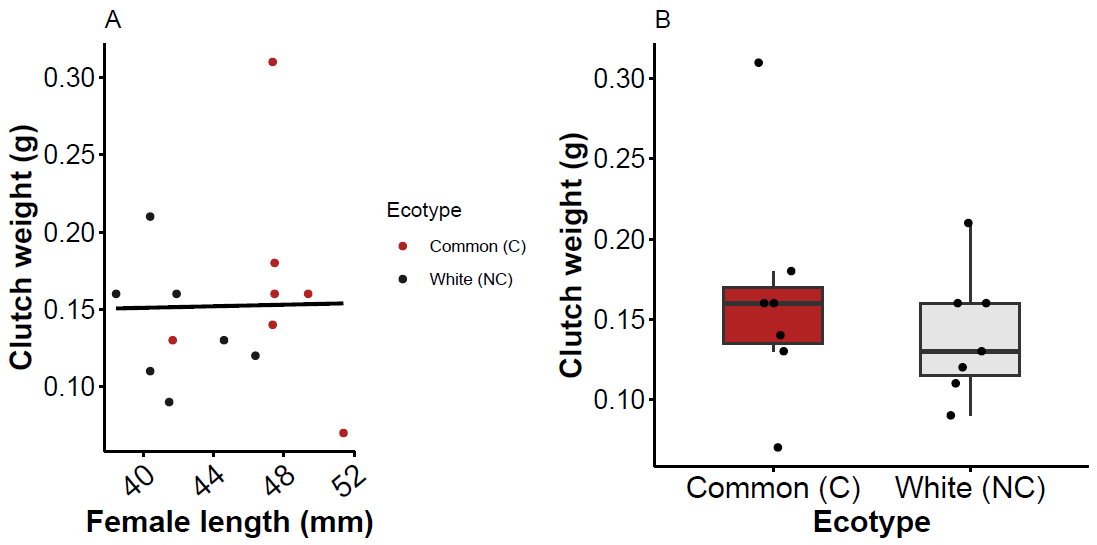


**Figure S4.** The number of eggs in a clutch (clutch size) is negatively associated with the size of the eggs.


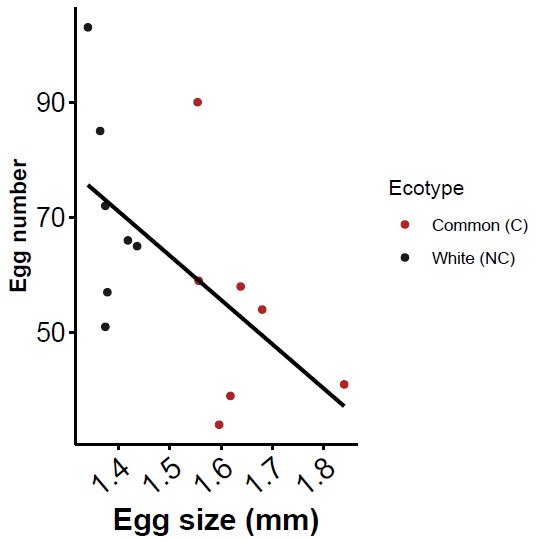

Supplement: Supplementary file 1 — Figure S1. Figure S2. Figure S3. Figure S4. [file ECE3-15-e70497-s001.docx]
